# Diagnostic performance of stress myocardial perfusion imaging for coronary artery disease: a systematic review and meta-analysis

**DOI:** 10.1007/s00330-012-2434-1

**Published:** 2012-04-19

**Authors:** Marcus C. de Jong, Tessa S. S. Genders, Robert-Jan van Geuns, Adriaan Moelker, M. G. Myriam Hunink

**Affiliations:** 1 Departments of Epidemiology and Radiology, Erasmus MC – University Medical Center Rotterdam, P.O. Box 2040, 3000 CA Rotterdam, The Netherlands; 2 Department of Radiology, Erasmus University Medical Center, Rotterdam, The Netherlands; 3 Department of Cardiology, Erasmus University Medical Center, Rotterdam, The Netherlands; 4 Department of Health Policy and Management, Harvard School of Public Health, Harvard University, Boston, USA

**Keywords:** Myocardial perfusion imaging, Diagnostic performance, Systematic review, Meta-analysis, Coronary artery disease

## Abstract

**Objectives:**

To determine and compare the diagnostic performance of stress myocardial perfusion imaging (MPI) for the diagnosis of obstructive coronary artery disease (CAD), using conventional coronary angiography (CCA) as the reference standard.

**Methods:**

We searched Medline and Embase for literature that evaluated stress MPI for the diagnosis of obstructive CAD using magnetic resonance imaging (MRI), contrast-enhanced echocardiography (ECHO), single-photon emission computed tomography (SPECT) and positron emission tomography (PET).

**Results:**

All pooled analyses were based on random effects models. Articles on MRI yielded a total of 2,970 patients from 28 studies, articles on ECHO yielded a sample size of 795 from 10 studies, articles on SPECT yielded 1,323 from 13 studies. For CAD defined as either at least 50 %, at least 70 % or at least 75 % lumen diameter reduction on CCA, the natural logarithms of the diagnostic odds ratio (lnDOR) for MRI (3.63; 95 % CI 3.26–4.00) was significantly higher compared to that of SPECT (2.76; 95 % CI 2.28–3.25; *P* = 0.006) and that of ECHO (2.83; 95 % CI 2.29–3.37; *P* = 0.02). There was no significant difference between the lnDOR of SPECT and ECHO (*P* = 0.52).

**Conclusion:**

Our results suggest that MRI is superior for the diagnosis of obstructive CAD compared with ECHO and SPECT. ECHO and SPECT demonstrated similar diagnostic performance.

**Key Points:**

• *MRI can assess myocardial perfusion*.

• *MR perfusion diagnoses coronary artery disease better than echocardiography or SPECT*.

• *Echocardiography and SPECT have similar diagnostic performance*.

• *MRI can save coronary artery disease patients from more invasive tests*.

• *MRI and SPECT show evidence of publication bias, implying possible overestimation*.

**Electronic supplementary material:**

The online version of this article (doi:10.1007/s00330-012-2434-1) contains supplementary material, which is available to authorized users.

## Introduction

Coronary artery disease (CAD) is one of the major causes of mortality and morbidity throughout the world [1]. The initial assessment of a patient with chest pain usually consists of a stress ECG (electrocardiogram). However, its diagnostic accuracy is low [2] compared to conventional coronary angiography (CCA), which is the reference standard for diagnosing CAD. On the other hand, CCA is an invasive technique and carries a small risk of complications [3, 4]. Myocardial perfusion imaging (MPI) is a non-invasive technique that is used clinically as a gatekeeper test before CCA.

MPI can be conducted using stress magnetic resonance imaging (MRI), contrast-enhanced echocardiography (ECHO), single-photon emission computed tomography (SPECT), positron emission tomography (PET) and, under development, computed tomography (CT). The only available extensive study directly comparing two techniques is the MR-IMPACT study [5], a multicentre randomised trial which found that MRI is superior to SPECT. Systematic reviews and meta-analyses have been published for most of the techniques but none of these reviews compare MPI techniques [6–10]. The comparability between these different meta-analyses is questionable mainly because of differences in publication period, searching the literature, selection of the evidence, and analysis of the data. Furthermore, studies with verification bias are often included in these reports which may have overestimated the sensitivity and underestimated the specificity of the tests considered. To overcome these problems a systematic review of different MPI techniques is required using the same selection criteria and methods of analysis for all techniques and excluding studies with (potential) verification bias, to make a fair comparison between these imaging tests.

The aim of this study was to determine and compare the diagnostic performance of stress MPI tests for the diagnosis of obstructive CAD, with conventional CCA as the reference standard. We performed the review according to the PRISMA statement for such reviews [11, 12].

## Materials and methods

### Search strategy

We searched Medline and Embase for English-language literature published between January 2000 and May 2011 evaluating the presence of obstructive CAD by stress perfusion imaging tests, namely MRI, contrast-enhanced ECHO, SPECT and PET. In this meta-analysis we focus on functional imaging tests evaluating perfusion as a measure of haemodynamically significant myocardial ischaemia as opposed to anatomical imaging tests, such as coronary CT angiography, which evaluates structural abnormalities of the coronary arteries. We limited the search to publications from 2000 onwards to include only studies that evaluated state-of-the-art MPI techniques. This may have introduced a selection bias with respect to SPECT, because many SPECT studies were published before 2000. To deal with this problem we compare our results with a review of meta-analyses of SPECT studies by Heijenbrok-Kal et al. [13]. CT was excluded because it is still being developed technically. Review articles were checked for potential additional studies. The search included keywords corresponding to the four index tests (MRI, ECHO, SPECT and PET), the reference test (CCA), the target condition (CAD) and diagnostic performance. We used numerous synonyms including both ‘text words’ and MeSH (Medical Subject Headings) terms to maximise the sensitivity of our search. See Appendix A in the Electronic Supplementary Material for a detailed description of the search strategy.

### Study selection

Two authors reviewed article titles and abstracts for eligibility. Discrepancies were resolved by consensus.

We included studies if they met all of the following criteria: (1) the study assessed diagnostic performance of stress perfusion MRI, stress perfusion contrast-enhanced ECHO, stress perfusion SPECT, or stress perfusion PET as a diagnostic test for CAD, (2) a prospective study design was used, (3) the study population consisted of known (previously diagnosed) or suspected adult CAD patients, (4) CCA was used as the reference standard test in all patients irrespective of the non-invasive test result, i.e. selective verification was not present, (5) obstructive CAD was defined as at least 1 vessel with at least 50 %, at least 70 % or at least 75 % lumen diameter reduction and, (6) absolute numbers of true positives (TP), false positives (FP), true negatives (TN) and false negatives (FN) were available at the patient level or could be derived adequately.

Studies were excluded if they met one of the following criteria: (1) the article was a review or meta-analysis, (2) patients had (suspected) acute coronary syndrome (ACS), (3) normal healthy volunteers or asymptomatic patients were included, (4) less than 30 patients were included (criterion to avoid TPs, FPs, TNs or FNs of zero), (5) (potentially) overlapping study populations were reported, (6) a very specific patient population (e.g. only patients with a heart transplant, left bundle branch block or aortic stenosis) was studied, (7) the study focused on in-stent or graft stenosis after percutaneous coronary intervention (PCI) or coronary artery bypass grafting (CABG).

### Data extraction

Two authors independently extracted data on author, journal, year of publication, technique used, country, hospital type, number of patients, mean age, percentage male, patient selection, brand of imaging device, magnetic field strength, radiotracer, contrast agent used, type of assessment (qualitative or quantitative), stressor used, CAD definition and the numbers of TP, FP, TN and FN. Discrepancies were resolved by consensus.

If a study reported pairs of sensitivities and specificities at different cut-off points, we extracted the pair with the highest sensitivity. When studies reported data for multiple CAD definitions (e.g. at least 50 % and at least 70 % stenosis), the highest sensitivity was used to calculate the overall estimates. This also applied when studies reported sensitivities and specificities for different observers.

### Quality assessment

We used a modified QUADAS checklist (quality assessment of studies of diagnostic performance included in systematic reviews) [14] to assess the quality of included studies. Two authors independently assessed the study quality of the included articles. Discrepancies were resolved by consensus.

### Statistical analysis and data synthesis

We analysed the data at the patient level using a bivariate random effects regression model [15]. The model assumes a binomial distribution of the within-study variability (variability between sensitivity and specificity within a study). The model furthermore assumes correlated normally distributed random effects between studies. The degree of correlation between the logit sensitivity and logit specificity corresponds to the inverse relation between sensitivity and specificity when the positivity criterion is varied. Additionally, meta-regression was performed to explore the effect of differences in patient selection and CAD disease definition, taking into account the possible interaction between differences in CAD disease definition and the techniques considered.

The data of each study were summarised in forest plots and summary estimates with a 95 % confidence interval of sensitivity and specificity for each imaging technique. Additionally, we summarised these numbers in receiver operator characteristic (ROC) spaces showing the summary estimates with a 95 % confidence region and a summary curve. To distinguish SPECT studies that used different protocols, we highlighted the studies that combined gated-SPECT with the use of ^99m^technetium as a radiotracer (Fig. 4). Similarly, MRI studies that included the assessment of delayed contrast enhancement were highlighted. Figures were created using Cochrane’s Review Manager (version 5, Copenhagen, Denmark).

To estimate the clinical utility of each technique we calculated the positive and negative likelihood ratios (LR + and LR−). The likelihood ratio is equivalent to the ratio of the likelihood of a certain test result in patients with the disease and the likelihood of the same test result in those without the disease. LR+ [= sensitivity/(1 − specificity)] describes the likelihood when the test is positive and LR− [= (1  − sensitivity)/specificity] describes the likelihood when the test is negative. To illustrate the clinical utility, we used the LRs to calculate post-test probabilities across the range of possible pre-test probabilities (Fig. 5).

Finally, we calculated the natural logarithm of the diagnostic odds ratio (lnDOR). The lnDOR represents an overall summary estimate of diagnostic performance. The diagnostic odds ratio (DOR) is the odds of positive test results in patients with disease compared to the odds of positive test results in those without disease which equals the ratio of the positive and negative likelihood ratios.

We also created funnel plots to assess the presence of publication bias. The funnel plot shows the DOR horizontally and the standard error of the log transformed DOR vertically. Publication bias usually occurs when negative publications (in our case studies with a low DOR) with a small sample size are not published. An asymmetric funnel plot, for example one with fewer studies in the lower left part of the graph, suggests the presence of publication bias.

The statistical software package SAS (Proc NLMIXED, SAS v9.2, Raleigh, NC, USA) was used for the analyses.

## Results

**Fig. 1:**
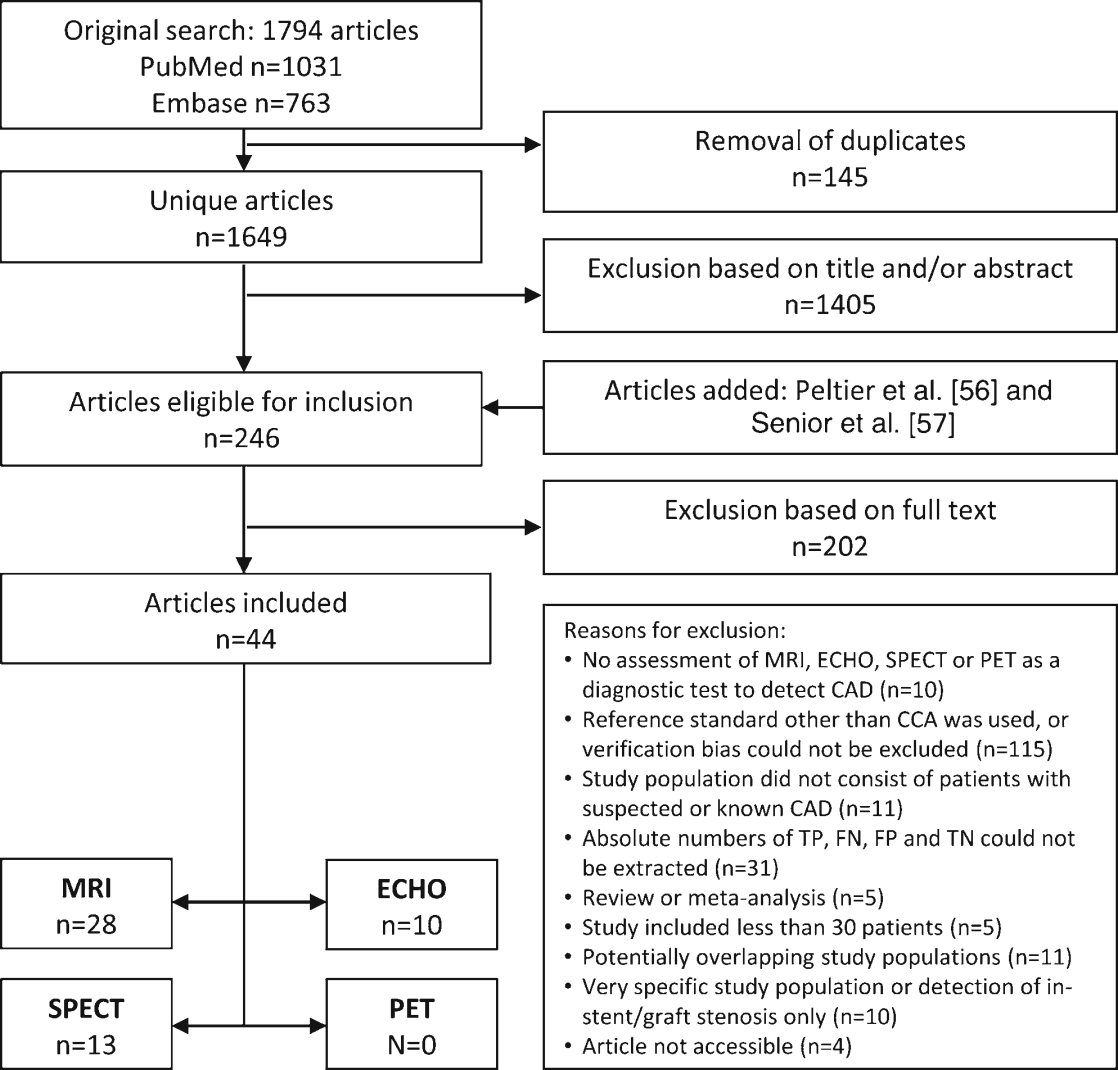
Flow chart of systematic literature search

Medline (PubMed) and Embase searches yielded 1,649 unique studies (Fig. 1). On the basis of title and abstract we excluded 1,405 articles. On the basis of the full text, we excluded 202 for various reasons detailed in Fig. 1. Review of the study characteristics shows considerable differences between the included studies (Table 1).

**Table 1:** Study characteristics

Author	Journal	Year	Technique	Country	Type	Patients (*n*)	Mean age	Age SD	% male	Patients^a^	Brand	Tesla	Perfusion tests	Assessment	Stressor	CAD definition
Arnold et al. [21]	JACC Cardiovasc Imaging	2010	MRI	UK	N	65	64	9	65	S&K	Siemens	3	Rest, stress, DE	Qualitative	Adenosine	≥50 %
Bernhardt et al. [22]	JACC Cardiovasc Imaging	2009	MRI	Germany and Canada	A	823	64	12	76	S&K	Philips	1.5	Stress, DE^b^	Qualitative	Adenosine	≥70 %
Cheng et al. [23]	J Am Coll Cardiol	2007	MRI	UK	A	61	64	8	75	S&K	Siemens	3	Rest, stress	Qualitative	Adenosine	≥50 %
Cury et al. [24]	Radiology	2006	MRI	Brasil	N	46	63	5	81	S&K	GE	1.5	Stress, DE	Qualitative	Dipyridamole	≥70 %
Donati et al. [25]	Am J Roentgenol	2010	MRI	Switzerland	A	65	64	9	81	S&K	Philips	1.5	Rest, stress, DE	Qualitative	Adenosine	>50 %
Doyle et al. [26]	J Cardiovasc Magn Reson	2003	MRI	USA	A	184	59	11	0	NS	Philips	1.5	Rest, stress	Semi-quantitative	Dipyridamole	≥70 %
Gebker et al. [27]	Radiology	2007	MRI	Germany	N	40	61	8	70	S&K	Philips	1.5	Rest, stress, DE	Qualitative	Adenosine	≥50 %
Gebker et al. [28]	Radiology	2008	MRI	Germany	N	101	62	8	70	S&K	Philips	3	Rest, stress, DE	Qualitative	Adenosine	≥50 %
Gebker et al. [29]	Int J Cardiol	2011	MRI	Germany	N	78	65	10	76	S&K	Philips	1.5	Rest, stress, DE	Qualitative	Dobutamine/atropine	≥70 %
Giang et al. [30]	Eur Heart J	2004	MRI	Switzerland	A	44	58	NA	78	S&K	GE	1.5	Stress	Semi-quantitative	Adenosine	≥50 %
Kawase et al. [31]	Osaka City Med J	2004	MRI	Japan	N	50	67	12	58	NS	Philips	1.5	Rest, stress	Qualitative	Nicorandil	≥70 %
Kitagawa, et al. [32]	Eur Radiol	2008	MRI	Japan	A	50	65	9	72	S&K	GE	1.5	Rest, stress, DE	Qualitative	ATP	≥50 %
Klein et al. [33]	J Cardiovasc Magn Reson	2008	MRI	Germany	N	51	60	10	65	NS	Philips	1.5	Rest, stress, DE	Qualitative	Adenosine	>50 %
Klein et al. [34]	JACC Cardiovasc Imaging	2009	MRI	UK and Germany	A	78	66	8	90	K	Philips	1.5	Stress, DE	Qualitative	Adenosine	>50 %
Klem et al. [35]	J Am Coll Cardiol	2006	MRI	USA	A	92	58	12	49	S	Siemens	1.5	Rest, stress, DE^b^	Qualitative	Adenosine	≥50 % and ≥70 % (≥50 LM)
Klumpp et al. [36]	Eur Radiol	2010	MRI	Germany	A	57	62	11	82	S&K	Siemens	3	Rest, stress, DE	Qualitative	Adenosine	>70 %
Krittayaphong et al. [37]	Int J Cardiovasc Imaging	2009	MRI	Thailand	A	66	61	12	58	S	Philips	1.5	Rest, stress	Semi-quantitative	Adenosine	≥50 %
Merkle et al. [38]	Heart	2007	MRI	Germany	A	228	61	11	79	S&K	Philips	1.5	Rest, stress, DE	Qualitative	Adenosine	>50 % and >70 %
Meyer et al. [39]	Eur Radiol	2008	MRI	Germany	A	60	59	10	63	S&K	Philips	3	Rest, stress, DE	Qualitative	Adenosine	≥70 %
Nagel et al. [40]	Circulation	2003	MRI	Germany	A	84	63	8	81	S	Philips	1.5	Rest, stress	Semi-quantitative	Adenosine	≥75 %^‡^
Paetsch et al. [41]	Circulation	2004	MRI	Germany	N	79	61	9	66	S&K	Philips	1.5	Rest, stress	Qualitative	Adenosine	>50 %
Pilz et al. [42]	Clin Res Cardiol	2006	MRI	Germany	A	171	62	12	63	S&K	GE	1.5	Rest, stress, DE	Qualitative	Adenosine	>70 %
Pingitore et al. [43]	Am J Cardiol	2008	MRI	Italy	A	93	61	NA	70	S&K	GE	1.5	Rest, stress	Quantitative	Dipyridamole	>50 %
Plein et al. [44]	Radiology	2005	MRI	UK	N	82	58	NA	74	S&K	Philips	1.5	Rest, stress	Semi-quantitative	Adenosine	>70 %
Plein et al. [45]	Eur Heart J	2008	MRI	Switzerland	A	51	59	10	76	S&K	Philips	1.5	Stress, DE	Qualitative	Adenosine	>50 %
Plein et al. [46]	Radiology	2008	MRI	Switzerland and UK	A	33	58	11	73	S&K	Philips	3	Stress, DE	Qualitative	Adenosine	>50 %
Stolzmann et al. [47]	Int J Cardiovasc Imaging	2010	MRI	Switzerland	A	65	64	10	87	NS	Philips	1.5	Rest, stress, DE	Semi-quantitative	Adenosine	>50 %
Takase et al. [48]	Jpn Heart J	2004	MRI	Japan	N	102	66	9	83	S&K	GE	1.5	Rest, stress, DE	Qualitative	Dipyridamole	>50 %
Author	Journal	Year	Technique	Country	Type	Patients (*n*)	Mean age	Age SD	% male	Patients	Brand	Contrast agent	Perfusion tests	Assessment	Stressor	CAD definition
Aggeli et al. [49]	Am J Hypertens	2007	ECHO	Greece	A	50	67	5	68	S	Philips	SonoVue	Rest, stress	Qualitative	Adenosine	≥50 %
Arnold et al. [21]	JACC Cardiovasc Imaging	2010	ECHO	UK	N	65	64	9	65	S&K	Philips	Optison	Rest, stress	Qualitative	Adenosine	≥50 %
Chiou et al. [50]	Can J Cardiol	2004	ECHO	Taiwan	N	132	67	11	75	S&K	Philips	PESDA	Rest, stress	Qualitative	Dobutamine	≥50 % (≥40 % LM)
Jeetley et al. [51]	J Am Coll Cardiol	2006	ECHO	UK	A/N	123	62	12	71	S&K	Philips	Sonazoid	Rest, stress	Semi-quantitative	Dipyridamole	≥70 %
Kowatsch et al. [52]	J Am Soc Echocardiogr	2007	ECHO	Brasil	A	54	60	9	61	S&K	Philips	PESDA	Rest, stress	Quantitative	Adenosine	>50 %
Lipiec et al. [53]	J Am Soc Echocardiogr	2008	ECHO	Poland	A	103	58	9	63	S&K	Siemens	Optison	Rest, stress	Qualitative	Dipyridamole or atropine	≥70 %
Miszalski-Jamka et al. [54]	Int J Cardiol	2009	ECHO	Poland	A	103	58	NA	80	S&K	Philips	Sonovue	Rest, stress	Qualitative	Exercise	≥50 %
Moir et al. [55]	J Am Soc Echocardiogr	2005	ECHO	Australia	A	79	56	NA	80	S&K	Philips	Definity	Rest, stress	Quantitative	Dipyridamole, exercise	≥50 %
Peltier et al. [56]	J Am Coll Cardiol	2004	ECHO	Belgium	A	35	62	10	71	S&K	Agilent Technologies	PESDA	Rest, stress	Quantitative	Dipyridamole	>70 %
Senior et al. [57]	Am Heart J	2004	ECHO	UK, Germany, Belgium	N	54	61^†^	NA	82	NS	Philips	Sonazoid	Rest, stress	Qualitative	Dipyridamole	>50 %
Author	Journal	Year	Technique	Country	Type	Patients (*n*)	Mean age	Age SD	% male	Patients	Brand	Radiotracer	Perfusion tests	Assessment	Stressor	CAD definition
Aggeli et al. [49]	Am J Hypertens	2007	SPECT	Greece	A	48	67	5	68	S	GE	^201^Tl	Rest, stress	Qualitative and qualitative	Adenosine	≥50 %
Astarita et al. [58]	J Hypertens	2001	SPECT	Italy	N	53	58	10	55	S	Picker	^201^Tl	Rest, stress	Qualitative	Exercise	≥50 %
Budoff et al. [59]	Acad Radiol	2007	SPECT	USA	A	30	54	9	70	NS	NS	^99m^Tc-MIBI	Rest, stress	Qualitative	Exercise	>70 % (>50 % LM)
Doyle et al. [26]	J Cardiovasc Magn Reson	2003	SPECT	USA	A	184	59	11	0	NS	ADAC	^99m^Tc-MIBI/^201^Tl	Rest, stress (gated)	Qualitative	Dipyridamole	≥70 %
Gonzalez et al. [60]	Rev Esp Med Nucl	2005	SPECT	Chile	A	145	60	12	68	S&K	GE	^201^Tl	Rest, stress	Qualitative	Exercise (*n* = 63), dipyridamole (*n* = 82)	≥50 % and ≥75 %
Jeetley et al. [51]	J Am Coll Cardiol	2006	SPECT	UK	A/N	123	62	12	71	S&K	NS	^99m^Tc-MIBI	Rest, stress	Semi-quantitative	Dipyridamole	≥70 %
Johansen et al. [61]	J Nucl Cardiol	2005	SPECT	Denmark	A	357	57	9	54	S	Picker	^201^Tl(rest) and ^99m^Tc-MIBI (stress)	Rest, stress (gated)	Semi-quantitative	Adenosine or dobutamine (*n* = 180), exercise stress test (*n* = 177)	≥50 %
Lipiec et al. [53]	J Am Soc Echocardiogr	2008	SPECT	Poland	A	103	58	9	63	S&K	GE	^99m^Tc-MIBI	Rest, stress	Semi-quantitative	Dipyridamole	≥70 %
Peltier et al. [56]	J Am Coll Cardiol	2004	SPECT	Belgium	A	35	62	10	71	S&K	GE	^99m^Tc-MIBI	Rest, stress	Qualitative	Dipyridamole	>70 %
Schepis et al. [62]	J Nucl Med	2007	SPECT	Switzerland	A	77	66	9	62	S	GE	^99m^Tc-tetrofosmin	Rest, stress (gated)	Semi-quantitative	Adenosine	≥50 %
Senior et al. [57]	Am Heart J	2004	SPECT	UK, Germany, Belgium	N	53	61^†^	NA	82	NS	Amersham Health	^99m^Tc-tetrofosmin	Rest, stress	Qualitative	Dipyridamole	>50 %
Yao et al. [63]	Nucl Med Commun	2000	SPECT	China	N	64	51	NA	95	S&K	Siemens	^99m^Tc-MIBI	Rest, stress	Qualitative	Exercise	≥50 %
Yeih et al. [64]	J Formos Med Assoc	2007	SPECT	Taiwan	A	51	63	9	0	S&K	GE	^201^Tl	Rest, stress	Qualitative	Dobutamine	≥50 %

*A* academic, *N* non-academic, *NS* not specified, *NA* not available, *PESDA* perfluorocarbon-exposed sonicated dextrose albumin, *S* suspected CAD only (and no history of myocardial infarction (MI), CABG or PCI), *S&K* patients with either suspected or known CAD, *K* known CAD, ^*99m*^ *Tc* ^99m^technetium, *-MIBI* sestamibi, ^*201*^ *Tl* ^201^thallium, *DE* delayed enhancement, *ATP* adenosine triphosphate, *LM* left main coronary artery

^†^Median; ^‡^area reduction

^a^If there was any uncertainty about the study population it was not classified as suspected and/or known CAD but rather as “not specified”

^b^In Klem et al. [35] and Bernhardt et al. [22] the index test outcome was initially based on delayed enhancement (DE) images, considering perfusion images when DE was negative

**Fig. 2:**
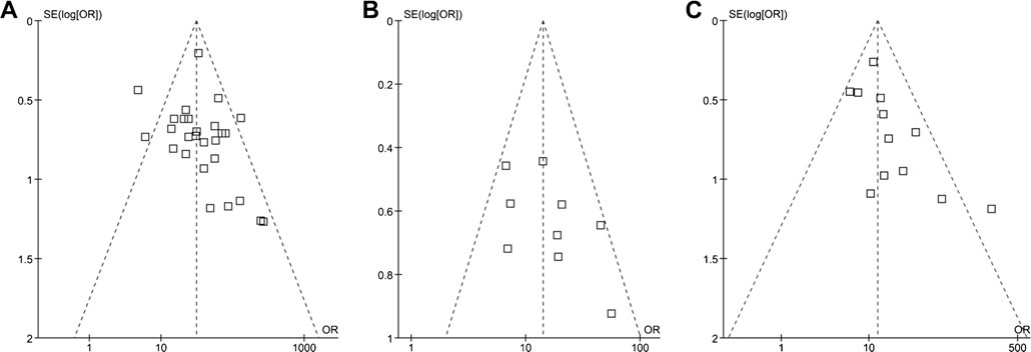
Funnel plots. The diagnostic odds ratio (DOR) on the *x*-axis is plotted against the standard error (SE) of the log(DOR) on the *y*-axis. A symmetrical distribution of studies indicates the absence of publication bias. An asymmetrical distribution with, for example, relatively more smaller studies with a positive result (in the *lower right part* of the plot) would suggest the presence of publication bias. In the ECHO funnel plot Peltier et al. [56], in the SPECT funnel plot Astarita et al. [58] and in the MRI funnel plot Donati et al. [25] are not included, because their respective DORs could not be calculated (0 false negatives or false positives). **a** MRI, **b** ECHO, **c** SPECT

**Fig. 3:**
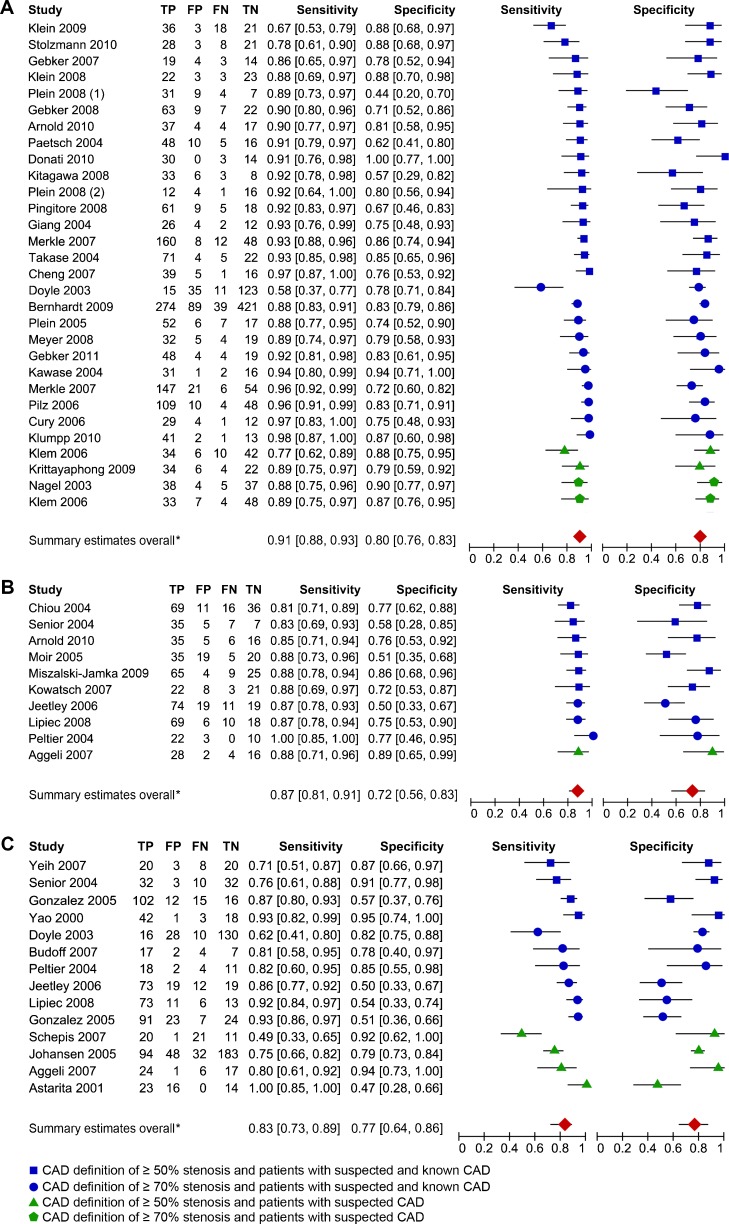
Forest plots. The data are sorted by suspected and known CAD versus suspected CAD and CAD definition of ≥50 % versus ≥70 % stenosis from lowest to highest sensitivity and data are reported at the patient level. **a** MRI, **b** ECHO, **c** SPECT. *When data were available for both CAD definitions (≥50 % and ≥70 %) the summary estimates only include data from CAD ≥70 % stenosis

Forty-four studies met the inclusion criteria. Articles on MRI yielded a total of 2,970 patients from 28 studies, articles on ECHO yielded a sample size of 795 from 10 studies, articles on SPECT yielded 1,323 from 13 studies. We could not include any PET studies, which is why PET was excluded from the analysis. The overview of the QUADAS checklist for all studies demonstrates some differences in terms of study quality (see Appendix B in the Electronic Supplementary Material). The funnel plots of MRI and SPECT suggest evidence for publication bias, whereas the funnel plot of ECHO shows no obvious evidence for publication bias (Fig. 2). The sensitivities and specificities of each study vary across studies with sample sizes ranging from 30 to 823 (Tables 2 and 3). The forest plots show the sensitivities and specificities of each study with their 95 % confidence intervals depicted as horizontal lines (Fig. 3), grouped by CAD definition and study population and then sorted by sensitivity.

**Table 2:** Source data for MRI, ECHO and SPECT

Author	Year	Technique	TP	FN	TN	FP	Sensitivity (%)	Specificity (%)	CAD definition
Arnold et al. [21]	2010	MRI	37	4	17	4	90.2	81.0	≥50 %
Bernhardt et al. [22]	2009	MRI	274	39	421	89	87.5	82.5	≥70 %
Cheng et al. [23]	2007	MRI	39	1	16	5	97.5	76.2	≥50 %
Cury et al. [24]	2006	MRI	29	1	12	4	96.7	75.0	≥70 %
Donati et al. [25]	2010	MRI	30	3	14	0	90.9	100	>50 %
Doyle et al. [26]	2003	MRI	15	11	123	35	57.7	77.8	≥70 %
Gebker et al. [27]	2007	MRI	19	3	14	4	86.4	77.8	≥50 %
Gebker et al. [28]	2008	MRI	63	7	22	9	90.0	71.0	≥50 %
Gebker et al. [29]	2011	MRI	48	4	19	4	92.3	82.6	≥70 %
Giang et al. [30]	2004	MRI	26	2	12	4	92.9	75.0	≥50 %
Kawase et al. [31]	2004	MRI	31	2	16	1	93.9	94.1	≥70 %
Kitagawa et al. [32]	2008	MRI	33	3	8	6	91.7	57.1	≥50 %
Klein et al. [33]	2008	MRI	22	3	23	3	88.0	88.5	>50 %
Klein et al. [34]	2009	MRI	36	18	21	3	66.7	87.5	>50 %
Klem et al. [35]	2006	MRI	34	10	42	6	77.3	87.5	≥50 %
			33	4	48	7	89.2	87.3	≥70 % (≥50 LM)
Klumpp et al. [36]	2010	MRI	40	1	14	2	97.6	87.5	>70 %
Krittayaphong et al. [37]	2009	MRI	34	4	22	6	89.5	78.6	≥50 %
Merkle et al. [38]	2007	MRI	160	12	48	8	93.0	85.7	>50 %
			147	6	54	21	96.1	72.0	>70 %
Meyer et al. [39]	2008	MRI	32	4	19	5	88.9	79.2	≥70 %
Nagel et al. [40]	2003	MRI	38	5	37	4	88.4	90.2	≥75 %
Paetsch et al. [41]	2004	MRI	48	5	16	10	90.6	61.5	>50 %
Pilz et al. [42]	2006	MRI	109	4	48	10	96.5	82.8	>70 %
Pingitore et al. [43]	2008	MRI	61	5	18	9	92.4	66.7	>50 %
Plein et al. [44]	2005	MRI	52	7	17	6	88.1	73.9	>70 %
Plein et al. [45]	2008	MRI	31	4	7	9	88.6	43.8	>50 %
Plein et al. [46]	2008	MRI	12	1	16	4	92.3	80.0	>50 %
Stolzmann et al. [47]	2010	MRI	28	8	21	3	77.8	87.5	>50 %
Takase et al. [48]	2004	MRI	71	5	22	4	93.4	84.6	>50 %
Aggeli et al. [49]	2007	ECHO	28	4	16	2	87.5	88.9	≥50 %
Arnold et al. [21]	2010	ECHO	35	6	16	5	85.4	76.2	≥50 %
Chiou et al. [50]	2004	ECHO	69	16	36	11	81.2	76.6	≥50 % (≥40 % LM)
Jeetley et al. [51]	2006	ECHO	74	11	19	19	87.1	50.0	≥70 %
Kowatsch et al. [52]	2007	ECHO	22	3	21	8	88.0	72.4	>50 %
Lipiec et al. [53]	2008	ECHO	69	10	18	6	87.3	75.0	≥70 %
Miszalski-Jamka et al. [54]	2009	ECHO	65	9	25	4	87.8	86.2	≥50 %
Moir et al. [55]	2005	ECHO	35	5	20	19	87.5	51.3	≥50 %
Peltier et al. [56]	2004	ECHO	22	0	10	3	100.0	76.9	>70 %
Senior et al. [57]	2004	ECHO	35	7	7	5	83.3	58.3	>50 %
Aggeli et al. [49]	2007	SPECT	24	6	17	1	80.0	94.4	≥50 %
Astarita et al. [58]	2001	SPECT	23	0	14	16	100.0	46.7	≥50 %
Budoff et al. [59]	2007	SPECT	17	4	7	2	81.0	77.8	>70 % (>50 % LM)
Doyle et al. [26]	2003	SPECT	16	10	130	28	61.5	82.3	≥70 %
Gonzalez et al. [60]	2005	SPECT	102	15	16	12	87.2	57.1	≥50 %
			91	7	24	23	92.9	51.1	≥75 %
Jeetley et al. [51]	2006	SPECT	73	12	19	19	85.9	50.0	≥70 %
Johansen et al. [61]	2005	SPECT	94	32	183	48	74.6	79.2	≥50 %
Lipiec et al. [53]	2008	SPECT	73	6	13	11	92.4	54.2	≥70 %
Peltier et al. [56]	2004	SPECT	18	4	11	2	81.8	84.6	>70 %
Schepis et al. [62]	2007	SPECT	32	10	32	3	76.2	91.4	≥50 %
Senior et al. [57]	2004	SPECT	20	21	11	1	48.8	91.7	>50 %
Yao et al. [63]	2000	SPECT	42	3	18	1	93.3	94.7	≥50 %
Yeih et al. [64]	2007	SPECT	20	8	20	3	71.4	87.0	≥50 %

**Table 3:**
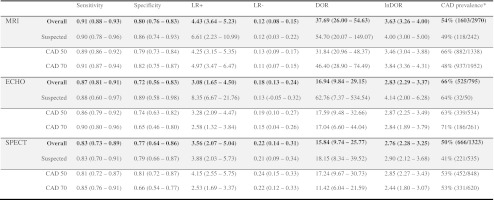
Measures of diagnostic performance for MRI, ECHO and SPECT, estimated using the bivariate random effects model

When data were available for both CAD definitions (≥50 % and ≥70 %) the overall estimates only include data from CAD ≥70 % stenosis

CAD 50 corresponds to the studies that defined obstructive CAD either as >50 % or ≥50 % stenosis

CAD 70 corresponds to the studies that defined obstructive CAD either as >70 %, ≥70 % or ≥75 % stenosis and studies that combined one of these with >50 % (or ≥50 %) stenosis in the left main coronary artery

“Suspected” refers to studies that only included patients with suspected CAD without a history of MI, PCI or CABG.

^a^The CAD prevalence defined by “CAD diagnosed by CCA” divided by the total sample size

**Fig. 4:**
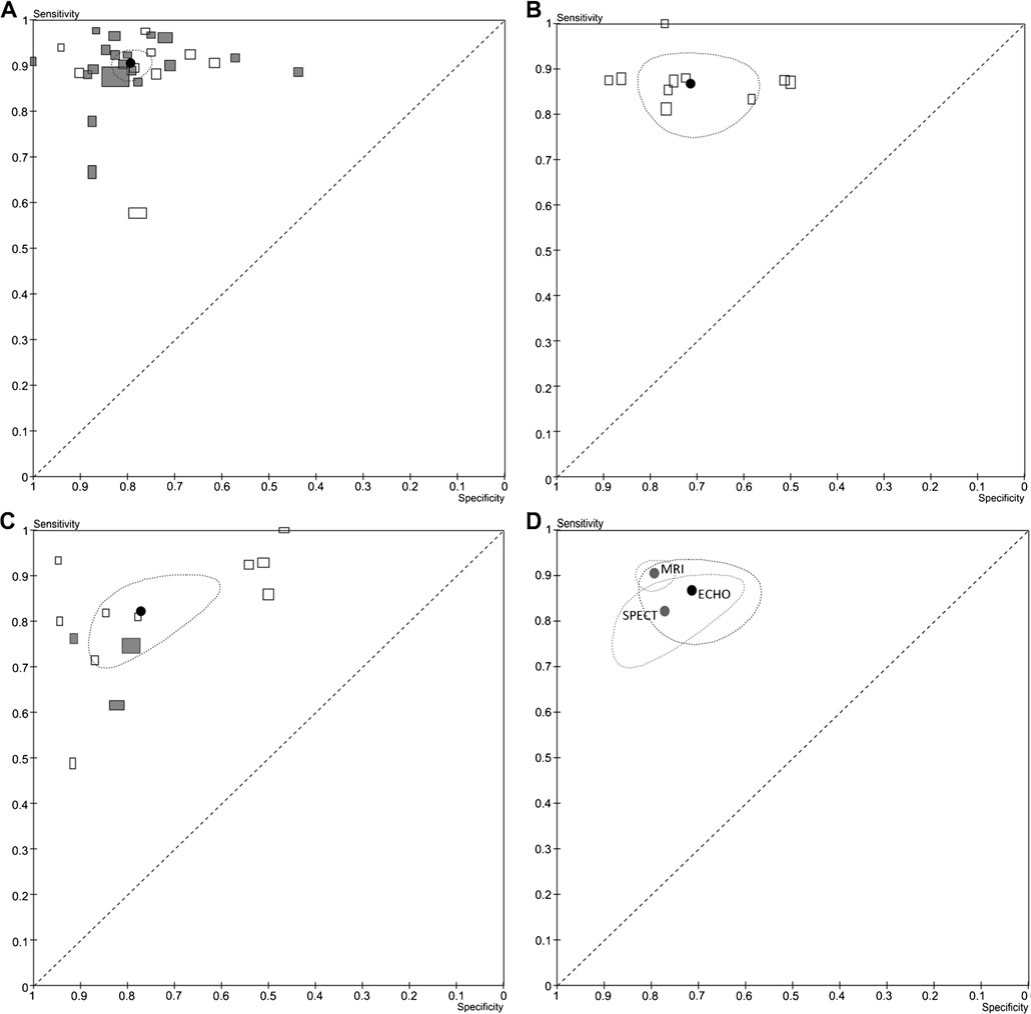
ROC space with summary estimates for each technique with 95 % confidence areas. This figure shows the diagnostic performance of studies relative to each other with specificity (plotted in reverse) on the *x*-axis and sensitivity on the *y*-axis. Perfect diagnostic accuracy is in the *upper left corner*, where sensitivity and specificity are both 1. **a** MRI, **b** ECHO, **c** SPECT, **d** All three techniques. The *grey rectangles* in **a** refer to the studies using delayed contrast enhancement and in **c** they refer to the studies using gated SPECT with radiotracer ^99m^Tc. The size of the *rectangles* corresponds with the inverse standard error of sensitivity and specificity, which correlates with the size of the study

Compared with coronary angiography the meta-analysis of the sensitivities and specificities of the different techniques (Table 3; Fig. 3) resulted for MRI in a sensitivity of 0.91 (95 % CI 0.88–0.93) and a specificity of 0.80 (95 % CI 0.76–0.83). Perfusion ECHO showed a sensitivity of 0.87 (95 % CI 0.81–0.91) and a specificity of 0.72 (95 % CI 0.56–0.83). SPECT demonstrated a sensitivity of 0.83 (95 % CI 0.73–0.89) and a specificity of 0.77 (95 % CI 0.64–0.86). The ROC spaces show the summary estimates for sensitivity and specificity of each technique two-dimensionally surrounded by its 95 % confidence area (Fig. 4). The sensitivity of MRI and SPECT differed significantly (*P* = 0.03). In terms of specificity, no significant differences were found.

We found no effect of CAD definition on the sensitivities (Table 3; *P* = 0.55). The disease definition greater than/at least 70 % stenosis compared to greater than/at least 50 % stenosis resulted in significantly lower specificities for SPECT (Table 3; *P* = 0.045), but no significant differences for ECHO (*P* = 0.39) and MRI (*P* = 0.51). Furthermore, we found no effect of CAD definition on the lnDORs of MRI (*P* = 0.24), ECHO (*P* = 0.96) and SPECT (*P* = 0.34) (Table 3).

Furthermore, MRI, ECHO and SPECT showed no significant differences in terms of sensitivity, specificity and lnDOR when comparing patients with suspected CAD without a prior history of CAD to patients with known or suspected CAD (all *P* values >0.05; Table 3).

We did not observe an association between the use of gated-SPECT in combination with ^99m^technetium as radiotracer and the diagnostic performance of SPECT (Fig. 4). MRI studies that assessed delayed contrast enhancement were associated with high sensitivities albeit with a wide range of specificities (Fig. 4).

**Fig. 5:**
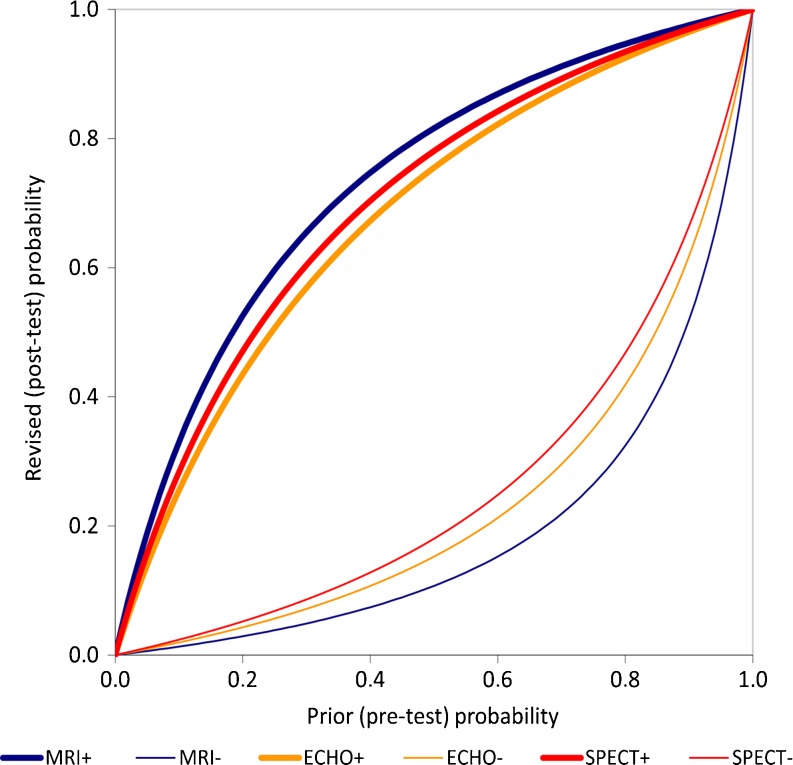
Revised probability of CAD. This figure shows the revised (post-test) probability of CAD (*y*-axis) as a function of prior (pre-test) probability (*x*-axis) of CAD for positive and negative MPI results, based on the likelihood ratios presented in Table 3 (overall analysis). MRI+, ECHO+ and SPECT+ represent the lines for a positive test result and MRI−, ECHO− and SPECT− represent the lines for a negative test result

The positive likelihood ratios (LR+) of MRI, ECHO and SPECT were 4.43 (95 % CI 3.64–5.23), 3.08 (95 % CI 1.65–4.50) and 3.56 (95 % CI 2.07–5.04) respectively (Table 3). The negative likelihood ratios (LR-) for MRI, ECHO, and SPECT were 0.12 (95 % CI 0.08–0.15), 0.18 (95 % CI 0.13–0.24) and 0.22 (95 % CI 0.14–0.31), respectively. Figure 5 illustrates the revised probability of CAD after a positive and negative test. The lnDORs of MRI, ECHO and SPECT were 3.63 (95 % CI 3.26–4.00), 2.83 (95 % CI 2.29–3.37) and 2.76 (95 % CI 2.28–3.25), respectively (Table 3). We found significantly higher lnDORs for MRI in comparison with SPECT (*P* = 0.006) and ECHO (*P* = 0.02). There was no significant difference between the lnDOR of SPECT and ECHO (*P* = 0.52).

## Discussion

In this systematic review and meta-analysis we compared the diagnostic performance of different stress MPI techniques. MRI showed the best diagnostic performance with the narrowest confidence intervals; the latter is explained by the large number of patients studied with MRI. We found a significantly higher sensitivity for MRI compared to SPECT and a significantly higher lnDOR for MRI compared to both ECHO and SPECT. In contrast to previous meta-analyses [9], we compared the different imaging techniques using the same search strategy and methods of analysing the data. Furthermore, we only included studies without verification bias.

In our review we paid special attention to the issue of verification bias. Sensitivity may be overestimated and specificity underestimated if patients with a positive test result are more likely to be verified with the reference standard test. Diagnostic odds ratios are generally not, or only minimally, affected by verification bias [16]. Underwood et al. [9] reviewed the diagnostic performance of SPECT and explained the overall low specificity (0.70–0.75 for high quality studies) of SPECT studies by verification bias. In their review of SPECT studies, Heijenbrok-Kal et al. [13] did not exclude studies with verification bias and demonstrated a sensitivity of 0.88 (95 % CI 0.87–0.90) and a specificity of 0.73 (95 % CI 0.69–0.74). By excluding studies with verification bias, we found a lower sensitivity of 0.83 (95 % CI 0.73–0.89), but a higher specificity of 0.77 (95 % CI 0.64–0.86). As pointed out above, the diagnostic odds ratios are less affected by verification bias and were the same for the previous and current review.

Nandalur et al. [7] and Hamon et al. [10] previously studied the diagnostic performance of myocardial perfusion MRI and found sensitivities of 91 % and 89 % respectively and specificities of 81 % and 80 % respectively, which is very similar to what we found. Unfortunately we could not include PET in the analysis, because no PET studies met our inclusion and exclusion criteria. Nandalur et al. [6] performed a meta-analysis of PET perfusion studies and they found a sensitivity of 0.92 and a specificity of 0.85. However, their analysis included studies with potential verification bias. Stress perfusion CT is an upcoming MPI technique, but we did not include this technique because of the low number of available studies and because perfusion CT is still in the technical development phase.

Other promising alternatives to CCA are non-invasive CT and MR coronary angiography. Schuetz et al. [17] compared CT and MR coronary angiography to CCA in a meta-analysis resulting in a sensitivity and specificity of respectively 0.97 and 0.87 for CT, and 0.87 and 0.70 for MR, suggesting that CT angiography has a better diagnostic performance compared to the MPI techniques analysed in this article. However, drawbacks of CT angiography are the use of iodinated contrast material which poses a small risk of idiosyncratic reactions and nephrotoxicity and the lack of functional information [18].

### Limitations

We focused on the diagnostic performance of MPI. However, an MPI examination can yield functional information as well (e.g. left ventricular function, presence of wall motion abnormalities, presence of scar tissue), rather than perfusion images alone. Our analysis does not take into account the possible impact of these parameters on the interpretation of the MPI test and the results of MRI are therefore likely to be even better than we estimated.

Also, it is important to note that in clinical practice a small proportion of patients will be unsuitable for MRI, either due to contraindications or claustrophobia. Likewise, an echocardiography procedure relies on an adequate acoustic window. Often, unsuitable patients were excluded from the original studies, which in turn could have resulted in an overestimation of the diagnostic performance in our analysis. Unfortunately, the included studies did not report sufficient information to explore these issues.

In the current review we included only studies that used the most advanced technology by searching for studies published from 2000 until 2011, which implies that some large landmark SPECT studies performed in the 1980s and 1990s were excluded from our analysis. A previously published comprehensive systematic review sheds light on the effect of this exclusion criterion [13]. In the previous review 103 SPECT studies with a total of 11,977 patients published between 1984 and 2002 were analysed. There is no overlap with the SPECT studies that we included. The diagnostic odds ratios for SPECT found in the previous review and in the current review are the same: they found an lnDOR of 2.8 (95 % CI 2.6–3.0) compared to our lnDOR of 2.8 (95 % CI 2.3–3.3).

The funnel plot for MRI and SPECT suggests that there is evidence of publication bias, which implies that our summary measures may be overestimated. Nevertheless, the overestimation applies to both MRI and SPECT. The funnel plot for ECHO does not suggest evidence of publication bias.

Heterogeneity across studies is a limitation of meta-analyses of diagnostic performance. Across studies differences exist with respect to imaging techniques, assessment methods, stressors, radiotracers, contrast media, CAD definition (lumen diameter reduction of at least 50 %, at least 70 % or at least 75 %), CAD prevalence, percentage male patients, patient inclusion criteria, setting and country. Although we were able to analyse the effect of using different CAD definitions and patient inclusion criteria, sample size limitations did not allow us to do subset analyses for the other cross-study variations. Due to chance there will always be variability between studies, but there may also be different types of biases influencing the results. We used a random effects model which adjusts the estimates and confidence intervals to account for between-study variations. Nevertheless, heterogeneity across studies remains an important limitation.

For calculation and precision purposes, we excluded studies with less than 30 patients. In this way, we minimised the number of studies with for example zero FPs or FNs. This exclusion criterion may have introduced a selection bias.

Another limitation of meta-analyses is the dependence on the level of detail reported in the original papers. For example, data on the individual territories were generally not available. Furthermore, most studies included a mix of known and suspected CAD patients or did not report the test characteristics for the subgroup of patients with suspected CAD without a prior history of MI, PCI or CABG. Therefore, our subgroup analysis of suspected CAD was limited due to a small sample size. Nevertheless, our analysis did suggest that the diagnostic performance of MPI tests is not substantially affected by including patients with known CAD.

Although our results show that all tests are reasonably accurate, the likelihood ratios suggest that neither one of them is suitable to rule out or rule in the presence of disease [19]. This can also be seen in Fig. 5, where the post-test probability after a positive test rarely exceeds 90 %, and the post-test probability of disease after a negative test may still be substantial. Since MPI is intended as a gatekeeper test, ruling out disease is more important than ruling in disease. MRI performs quite well in this respect with an LR− of 0.12 (0.08–0.15). SPECT and ECHO demonstrate less favourable LRs (Table 3).

The reference standard test for diagnosing CAD is CCA. Innovative technological developments in diagnosing CAD are most often compared with CCA. The limitation of CCA is that it evaluates the lumen diameter reduction of the coronary arteries, but for instance a 50 % vessel diameter reduction does not always result in the same reduction in blood flow and does not necessarily lead to myocardial ischaemia. There are alternative techniques such as fractional flow reserve (FFR) that measure the pressure difference across a coronary stenosis. It is even possible that the imaging techniques we evaluated are better diagnostic tools than CCA to begin with, since they measure myocardial perfusion which is the physiological basis of myocardial function. Thus, the less than perfect sensitivity and specificity could in part be attributed to imperfections of CCA instead of the limitations of perfusion imaging.

### Clinical implications

The results of our systematic review and meta-analysis suggest that MRI is superior to ECHO and SPECT in diagnosing CAD. This statement is strengthened firstly by the findings of the MR-IMPACT study [5]—a multicentre randomised trial—which suggested that MRI is superior to SPECT and secondly by the findings of the EuroCMR registry [20], which demonstrated that in patients who underwent stress MRI for the diagnostic workup of suspected CAD, invasive angiography could be avoided in nearly one-half of the patients. All in all, the results suggest that stress perfusion MRI is potentially useful as a gatekeeper test before CCA in patients with low to intermediate prior probability of CAD but this needs to be confirmed with a comparative cost-effectiveness analysis. Furthermore, more research of the diagnostic performance of stress perfusion ECHO, PET and CT is required to evaluate their clinical usefulness.

In conclusion, our results suggest that stress perfusion MRI is superior for the diagnosis of obstructive CAD compared to stress perfusion contrast-enhanced echocardiography and SPECT, and that echocardiography and SPECT are similar in terms of diagnostic performance.

## Electronic supplementary material

Below is the link to the electronic supplementary material.

ESM 1 (DOC 187 kb)
