# Supplementary material for: Diagnostic performance of stress myocardial perfusion imaging for coronary artery disease: a systematic review and meta-analysis
Source: Eur Radiol. 2012 Apr 19;22(9):1881–95. doi: 10.1007/s00330-012-2434-1 (PMC3411304; doi:10.1007/s00330-012-2434-1)
Supplement: Supplementary file 1 — (DOC 187 kb) [file 330_2012_2434_MOESM1_ESM.doc]

**APPENDICES**

**Appendix A. Medline and Embase search**

*Index test MRI (1)*

"Magnetic Resonance Imaging"[Mesh] OR ("magnetic" AND "resonance"[tw]) OR NMR OR MRI[tw] OR MR[tw]

*Index test PET (2)*

"Positron-Emission Tomography"[Mesh] OR PET[tw] OR (((positron[tw] AND emission[tw]) OR positron-emitt*[tw]) AND tomograph*[tw]) OR rubidium[tw] OR nitrogen[tw] OR deoxyglucose[tw] OR FDG[tw] OR N-13[tw] OR N13[tw] OR 82rb[tw]

*Index test SPECT (3)*

("Tomography, Emission-Computed, Single-Photon"[Mesh] OR "Myocardial Perfusion Imaging"[Mesh] OR ((CT[tw] OR CAT[tw] OR (comput*[tw] AND tomograph*[tw])) AND single[tw] AND photon[tw]) OR SPECT[tw] OR SPET[tw] OR (myocard*[tw] AND perfusion[tw] AND imag*[tw]) OR (myocard*[tw] AND scintigraph*[tw]))

*Index test ECHO (4)*

("Ultrasonography"[Mesh] OR ultrasound[tw] OR ultrason*[tw] OR echocardio*[tw]) AND (perfusion[tw] OR "Perfusion Imaging"[Mesh])

*Stressor – index tests (5)*

dipyridamole[tw] OR dobutamine[tw] OR adenosine[tw] OR nicorandil[tw] OR stress*[tw] OR perfusion[tw] OR (blood[tw] AND flow[tw]) OR pharmacolog*[tw]

*Target condition (6)*

"Coronary Disease"[Mesh] OR (coronary[tw] AND (disease[tw] OR stenos*[tw])) OR “Angina Pectoris”[Mesh] OR angina[tw] OR (chest[tw] AND pain[tw]) OR "Myocardial Infarction"[Mesh] OR MI[tw] OR (myocardial[tw] AND infarction*[tw])

*Reference test (7)*

“Coronary Angiography”[Mesh] OR (coronary[tw] AND cathether*[tw]) OR angiograph*[tw] OR angiogram[tw] OR "Fractional Flow Reserve, Myocardial"[Mesh] OR FFR[tw] OR (fractional[tw] AND flow[tw] AND reserve[tw])

*Diagnostic keywords (8)*

"sensitivity and specificity"[Mesh] OR sensitivity[tw] OR specificity[tw] OR "predictive value of tests"[Mesh] OR "ROC Curve"[Mesh] OR roc*[tw] OR sroc[tw] OR "receiver operating characteristic"[tw] OR "receiver operator characteristic"[tw] OR "pre-test odds"[tw] OR "pretest odds"[tw] OR (pre-test[tw] AND probabilit*[tw]) OR (pretest[tw] AND probabilit*[tw]) OR "post-test odds"[tw] OR "posttest odds"[tw] OR ("post test"[tw] AND probabilit*[tw]) OR (posttest[tw] AND probabilit*[tw]) OR (likelihood[tw] AND ratio*[tw]) OR ("positive predictive"[tw] AND value*[tw]) OR ("negative predictive"[tw] AND value*[tw]) OR (false[tw] AND negative*[tw]) OR (false[tw] AND positive*[tw]) OR (true[tw] AND negative*[tw]) OR (true[tw] AND positive*[tw]) OR misdiagnosis[tw] OR misdiagnoses OR "Diagnostic Errors"[Mesh] OR (diagnost*[tw] AND (accuracy[tw] OR error*[tw] OR efficacy[tw]))

*Limits (9)*

(English[lang] NOT ((Animals[Mesh] NOT Humans[Mesh]) OR Editorial[Publication type] OR Comment[Publication type] OR Letter[Publication type] OR Case Reports[Publication type])

*Final Medline search*

(1 OR 2 OR 3 OR 4) AND 5 AND 6 AND 7 AND 8 AND 9

The Embase search was the same as the Medline search, but with only ‘text words’ and excluding Medline studies.

Appendix B. The quality checklist

| Author | Year | 1 | 2 | 3 | 4* | 5 | 6 | 7 | 8 | 9 | 10 | 11 | 12 | 13 | 14 | 15 | 16 |
| --- | --- | --- | --- | --- | --- | --- | --- | --- | --- | --- | --- | --- | --- | --- | --- | --- | --- |
| Aggeli, et al. [49] | 2007 | N | Y | Y | <1 month | Y | Y | Y | Y | Y | Y | U | N | Y | N | Y | N |
| Arnold, et al. [21] | 2010 | Y | Y | Y | <2 weeks | Y | Y | Y | Y | Y | Y | U | N | Y | Y | Y | N |
| Astarita, et al. [58] | 2001 | N | Y | Y | <15 days | Y | Y | Y | Y | Y | Y | Y | N | N | N | Y | N |
| Bernhardt, et al. [22] | 2009 | Y | Y | Y | <2 weeks (8±5 days) | Y | Y | Y | Y | Y | Y | Y | N | Y | Y | Y | N |
| Budoff, et al. [59] | 2007 | Y | Y | Y | < 1 month | Y | Y | Y | Y | Y | Y | Y | U | N | N | U | N |
| Cheng, et al. [23] | 2007 | Y | Y | Y | <2 weeks | Y | Y | Y | Y | Y | Y | U | N | Y | Y | Y | N |
| Chiou, et al. [50] | 2004 | Y | Y | Y | <7 days | Y | Y | Y | Y | Y | Y | Y | U | Y | Y | U | N |
| Cury, et al. [24] | 2006 | Y | Y | Y | <2 weeks | Y | Y | Y | Y | Y | Y | Y | N | Y | N | Y | Y |
| Donati, et al. [25] | 2010 | Y | Y | Y | < median of 8 days | Y | Y | Y | Y | Y | Y | Y | N | U | Y | Y | N |
| Doyle, et al. [26] | 2003 | N | N | Y | NS | Y | Y | Y | Y | Y | U | U | U | Y | Y | U | N |
| Gebker, et al. [27] | 2007 | Y | Y | Y | <24 hours | Y | Y | Y | Y | Y | Y | U | N | Y | Y | Y | Y |
| Gebker, et al. [28] | 2008 | Y | Y | Y | <48 hours | Y | Y | Y | Y | Y | Y | Y | N | Y | Y | Y | Y |
| Gebker, et al. [29] | 2011 | Y | Y | Y | <90 days | Y | Y | Y | Y | Y | Y | U | N | Y | Y | U | N |
| Giang, et al. [30] | 2004 | Y | Y | Y | <30 days | Y | Y | Y | Y | Y | Y | Y | N | Y | Y | Y | N |
| Gonzalez, et al. [60] | 2005 | Y | N | Y | < a mean of 27 days (range: 1 to  180) | Y | Y | Y | Y | Y | Y | U | N | N | N | Y | N |
| Jeetley, et al. [51] | 2006 | Y | N | Y | 4 weeks | Y | Y | Y | U | Y | Y | Y | N | N | N | Y | N |
| Johansen, et al. [61] | 2005 | Y | Y | Y | <3 month | Y | Y | Y | Y | Y | Y | Y | N | N | Y | Y | N |
| Kawase, et al. [31] | 2004 | Y | Y | Y | <1 weeks | Y | Y | Y | Y | Y | Y | U | N | N | N | Y | N |
| Kitagawa, et. Al. [32] | 2008 | Y | Y | Y | <14 days | Y | Y | Y | Y | Y | Y | Y | N | N | N | Y | Y |
| Klein, et al. [33] | 2008 | Y | Y | Y | <24 hours | Y | Y | Y | Y | N | Y | Y | N | Y | N | Y | Y |
| Klein, et al. [34] | 2009 | N | Y | Y | <24 hours | Y | Y | Y | Y | Y | Y | Y | U | Y | Y | U | N |
| Klem, et al. [35] | 2006 | Y | Y | Y | <24 hours | Y | Y | Y | Y | Y | Y | Y | N | Y | Y | Y | N |
| Klumpp, et al. [36] | 2010 | Y | Y | Y | NS | Y | Y | Y | Y | Y | U | U | N | Y | N | Y | N |
| Kowatsch, et al. [52] | 2007 | Y | Y | Y | <1 month | Y | Y | Y | Y | Y | Y | Y | N | Y | N | Y | N |
| Krittayaphong et. Al. [37] | 2009 | Y | Y | Y | <1 weeks | Y | Y | Y | Y | Y | Y | Y | U | N | Y | U | Y |
| Lipiec, et al. [53] | 2008 | Y | Y | Y | <14 days | Y | Y | Y | Y | Y | Y | Y | N | Y | N | Y | N |
| Merkle, et al. [38] | 2007 | Y | Y | Y | <4 weeks | Y | Y | Y | Y | Y | Y | Y | N | Y | Y | Y | N |
| Meyer, et al. [39] | 2008 | Y | Y | Y | <28 days | Y | Y | Y | Y | Y | Y | U | U | Y | N | U | N |
| Miszalski-Jamka, et al. [54] | 2008 | Y | N | Y | <15 days | Y | Y | Y | Y | Y | Y | Y | N | N | N | Y | N |
| Moir, et al. [55] | 2005 | Y | Y | Y | NS | Y | Y | Y | Y | Y | Y | Y | N | Y | N | Y | N |
| Nagel, et al. [40] | 2003 | Y | Y | Y | NS | Y | Y | Y | Y | Y | Y | Y | U | Y | Y | U | N |
| Paetsch, et al. [41] | 2004 | Y | Y | Y | NS | Y | Y | Y | Y | Y | Y | Y | U | Y | N | U | N |
| Peltier et al. [56] | 2004 | Y | Y | Y | NS | Y | Y | Y | Y | Y | Y | U | N | Y | N | Y | N |
| Pilz, et al. [42] | 2006 | Y | Y | Y | NS | Y | Y | Y | Y | Y | Y | U | U | Y | Y | U | N |
| Pingitore, et al. [43] | 2008 | Y | Y | Y | <15 days | Y | Y | Y | Y | Y | U | Y | U | Y | Y | U | N |
| Plein, et al. [44] | 2005 | Y | Y | Y | < mean of 4.3 days ±12 | Y | Y | Y | Y | Y | Y | Y | N | U | Y | Y | Y |
| Plein, et al. [45] | 2008 | Y | Y | Y | <median of 4 days (interquartile  range 6) | Y | Y | Y | Y | Y | Y | Y | N | Y | Y | Y | U |
| Plein, et al. [46] | 2008 | Y | N | Y | <14 days | Y | Y | Y | Y | Y | Y | Y | N | Y | Y | Y | Y |
| Schepis, et al. [62] | 2007 | Y | Y | Y | <2 weeks | Y | Y | Y | Y | Y | Y | Y | U | Y | N | U | N |
| Senior, et. al. [57] | 2004 | Y | Y | Y | <4 weeks | Y | Y | Y | Y | Y | Y | U | U | N | N | U | N |
| Stolzmann,et al. [47] | 2010 | Y | Y | Y | <14 days | Y | Y | Y | Y | Y | Y | Y | U | U | Y | U | N |
| Takase, et al. [48] | 2004 | Y | Y | Y | <1 month | Y | Y | Y | Y | Y | Y | U | N | N | N | Y | N |
| Yao, et al. [63] | 2000 | Y | N | Y | <3 weeks | Y | Y | Y | Y | Y | Y | U | U | N | N | U | N |
| Yeih, et al. [64] | 2007 | N | Y | Y | <2 weeks | Y | Y | Y | Y | Y | Y | U | U | N | N | U | N |

Y= yes; N = no; U = unclear; NS = not specified

1: Was the spectrum of patients representative of the patients who will receive the test in practice? 2: Were selection criteria clearly described? 3: Is the reference standard likely to correctly classify the target condition? 4: Is the time period between reference standard and index test short enough to be reasonably sure that the target condition did not change between the two tests?* 5: Did the whole sample or a random selection of the sample, receive verification using a reference standard of diagnosis? 6: Did patients receive the same reference standard regardless of the index test result? 7: Was the reference standard independent of the index test (i.e. the index test did not form part of the reference standard)? 8: Was the execution of the index test described in sufficient detail to permit replication of the test? 9: Was the execution of the reference standard described in sufficient detail to permit its replication? 10: Were the index test results interpreted without knowledge of the results of the reference standard? 11: Were the reference standard results interpreted without knowledge of the results of the index test? 12: Were the same clinical data available when test results were interpreted as would be available when the test is used in practice? 13: Were uninterpretable/intermediate test results reported? 14: Were withdrawals from the study explained? 15: Were the scans read blind to clinical data? 16: Was the expertise of the clinician assessing the results of the diagnostic tests reported?

*The time period between the index and reference tests as reported is tabulated to allow the reader to make his/her own judgment on this issue.
